# High Genomic Identity between Clinical and Environmental Strains of *Herbaspirillum frisingense* Suggests Pre-Adaptation to Different Hosts and Intrinsic Resistance to Multiple Drugs

**DOI:** 10.3390/antibiotics10111409

**Published:** 2021-11-18

**Authors:** Willian Klassen Oliveira, Hugo Leonardo Ávila, Michelle Zibeti Tadra, Rodrigo Luiz Cardoso, Cyntia Maria Teles Fadel-Pichet, Emanuel Maltempi de Souza, Fábio de Oliveira Pedrosa, Helisson Faoro

**Affiliations:** 1 Laboratory for Applied Science and Technology in Health, Instituto Carlos Chagas, FIOCRUZ Paraná, Algacyr Munhoz Mader Street, 3775, Curitiba 81350-010, Brazil; willianklasoli@gmail.com (W.K.O.); hugoavila@protonmail.com (H.L.Á.); 2 Graduation Program in Biofinformatics, Universidade Federal do Paraná, Curitiba 81520-260, Brazil; 3 Department of Biochemistry and Molecular Biology, Universidade Federal do Paraná, Curitiba 81531-980, Brazil; mzitadrasfeir@gmail.com (M.Z.T.); luiz.grabarski@gmail.com (R.L.C.); souzaem@ufpr.br (E.M.d.S.); fpedrosa@ufpr.br (F.d.O.P.); 4 Department of Clinical Analyses, Universidade Federal do Paraná, Curitiba 80060-240, Brazil; fpicheth@ufpr.br

**Keywords:** *H. frisingense*, AMR, host–pathogen interaction, genome comparison

## Abstract

The genus *Herbaspirillum* is widely studied for its ability to associate with grasses and to perform biological nitrogen fixation. However, the bacteria of the *Herbaspirillum* genus have frequently been isolated from clinical samples. Understanding the genomic characteristics that allow these bacteria to switch environments and become able to colonize human hosts is essential for monitoring emerging pathogens and predicting outbreaks. In this work, we describe the sequencing, assembly, and annotation of the genome of *H. frisingense* AU14559 isolated from the sputum of patients with cystic fibrosis, and its comparison with the genomes of the uropathogenic strain VT-16–41 and the environmental strains GSF30, BH-1, IAC152, and SG826. The genes responsible for biological nitrogen fixation were absent from all strains except for GSF30. On the other hand, genes encoding virulence and host interaction factors were mostly shared with environmental strains. We also identified a large set of intrinsic antibiotic resistance genes that were shared across all strains. Unlike other strains, in addition to unique genomic islands, AU14559 has a mutation that renders the biosynthesis of rhamnose and its incorporation into the exopolysaccharide unfeasible. These data suggest that *H. frisingense* has characteristics that provide it with the metabolic diversity needed to infect and colonize human hosts.

## 1. Introduction

The genus *Herbaspirillum* belongs to the beta class of the phylum Proteobacteria. Thirteen species are currently assigned to this genus; they have been isolated from various environments, including water [1], the rhizosphere [2], and plant tissues [3]. Three species of this genus, *Herbaspirillum frisingense, Herbaspirillum seropedicae*, and *Herbaspirillum rubrisubalbicans*, are described as nitrogen-fixing bacteria. These bacteria can convert atmospheric nitrogen (N_2_) into ammonium ions (NH^4+^), a form that can be assimilated by plants, through the nitrogenase enzyme complex encoded by *nifHDK* genes [4]. These bacteria establish an endophytic association with plants of economic interest, such as wheat, corn, sugarcane, and sorghum, thereby promoting the growth of these plants through nitrogen fixation in addition to the secretion of phytohormones such as indoleacetic acid [4].

The first identification of a clinical strain belonging to the genus *Herbaspirillum* occurred in 1996 when bacteria previously described as *Pseudomonas* were reclassified as *Herbaspirillum* [2]. Using techniques such as DNA–DNA hybridization and labeled probes, they identified 19 clinical isolates as belonging to the genus *Herbaspirillum*. These isolates originated in Germany, Sweden, and the United States, the oldest isolate being identified in 1968 [2]. Recently, isolates of the genus *Herbaspirillum* have been found more frequently in humans, especially in samples from immunocompromised patients with cancer, sepsis, cystic fibrosis, and pneumonia not related to cystic fibrosis [5,6,7,8,9,10,11,12,13,14,15]. The first clinical isolate of *H. frisingense* to undergo genomic sequencing was strain VT-16–41, which was isolated from a patient with a urinary tract infection [16]. In another study, Spilker et al. (2008) [17] identified 28 strains of the genus *Herbaspirillum* in sputum from patients with cystic fibrosis. Phylogenetic analysis showed that these strains represented various species of this genus; one, an isolate of *H. frisingense*, was designated as strain AU14559. However, it is not entirely clear whether *Herbaspirillum* bacteria are opportunistic, and thus only occasionally found in clinical samples, or whether they are pathogens that can cause secondary infections and worsen the condition of patients.

In addition to strain VT-16–41, the *H. frisingense* genomes deposited in public databases include those of three other strains identified as GSF30, IAC152, and cluster_DBSCAN_round4_6; all of these are environmental in origin. GSF30 is the type strain of the species and has been isolated from plants of the genus *Miscanthus* in southern Germany [4]. IAC152 has been isolated from sugarcane roots [18]. The cluster_DBSCAN_round4_6 genome has been sequenced and assembled from the microbiome associated with the eggs of the beetle *Lagria villosa*, and thus has not been isolated [19]. In this work, we describe the genome of *H. frisingense* AU14559 isolated from sputum obtained from patients with cystic fibrosis and its comparison with the genomes of other strains of this species for which genome sequence data are available in public databases. The aim of this work was to identify the genomic characteristics that have allowed this species to infect humans.

## 2. Results

### 2.1. Genome of H. frisingense AU14559

The genome assembly of *H. frisingense* AU14559 was performed based on data obtained through the NGS MiSeq and Ion Proton DNA sequencing platforms. Different assemblers were used to find the best *ab initio* assembly. The genome assemblies of *H. frisingense* AU14559 were evaluated using the QUAST program; the assembly from SPAdes yielded the best results and was chosen for further analysis. This assembly had 42 contigs and an N50 of 492 kb. After automatic gap closing using the FGAP tool, seven contigs remained. These were manually closed based on comparisons with the genome of *H. frisingense* GSF30 until a single contig representing the entire genome was obtained. The structure and reliability of the genome was checked by mapping back the reads in the assembly. No uncovered regions or inconsistencies between the paired-end reads were found. The closed genome of *H. frisingense* AU14559 consists of a single DNA molecule of 5,401,391 bp with 63.1% GC content; it contains 3 rRNA operons, encodes 59 tRNAs and 23 ncRNAs, and contains 4791 protein-coding genes covering 87.5% of the total genome size.

### 2.2. Identification of Publicly Available H. frisingense Genomes

**Figure 1:**
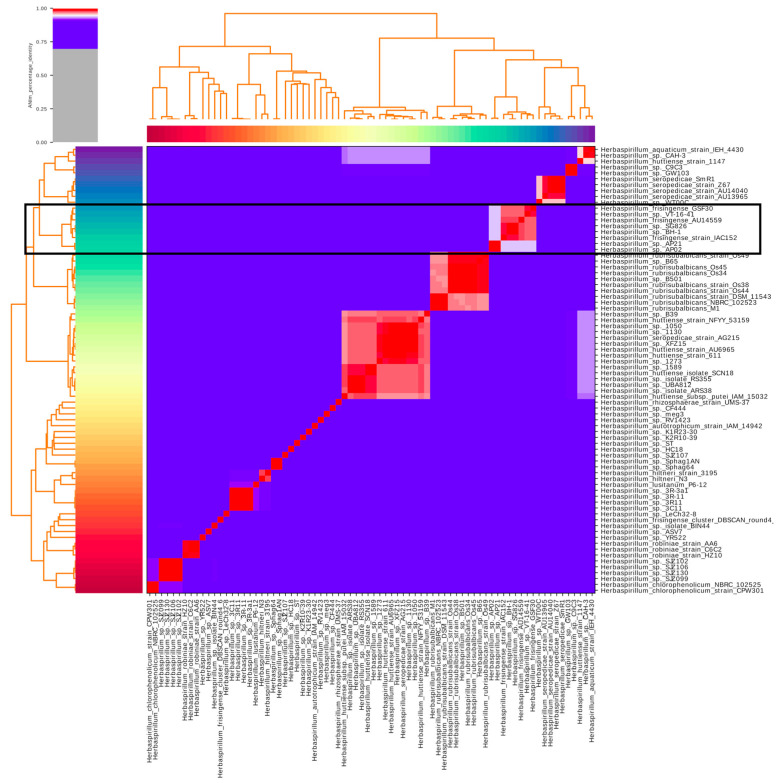
Average nucleotide identity heatmap (ANI) among all genomes of the genus *Herbaspirillum* deposited at NCBI. The ANIs were calculated by the pyani pipeline after aligning the regions present in all genomes of the genus *Herbaspirillum* through the Mummer. Genomes with ANI higher than 95% can be considered to belong to the same species; this identified the AU14559 isolate, as well as the strains *Herbaspirillum* sp.BH-1 and *Herbaspirillum* sp.SG826, as belonging to the species *Herbaspirillum frisingense*.

The *H. frisingense* strain AU14559 was named because of the high identity of the 16S rRNA gene with *H. frisingense* GSF30 [17]. To confirm that strain AU14559 belongs to the *H. frisingense* species and to identify other potential genomes of the species, we calculated the average nucleotide identity (ANI) between the genome of strain AU14559 and all other genomes identified as genus *Herbaspirillum* in GenBank (*n* = 76). In agreement with the result obtained by 16S rRNA gene comparison, the ANI calculation positioned the AU14559 strain genome in a cluster formed by the bacterial genomes of the *H. frisingense* species (Figure 1). Specifically, AU14559 was placed in a group along with *H. frisingense* GSF30 and *H. frisingense* VT-16–41. The genomic group of *H. frisingense* included three other genomes: *H. frisingense* IAC152, *Herbaspirillum* sp. BH-1, and *Herbaspirillum* sp. SG826. Two other genomes, *Herbaspirillum* sp. AP02 and *Herbaspirillum* sp. AP21, were also positioned close to the genomic group of *H. frisingense*; however, the ANI values for these genomes were below the 95% identity cutoff for inclusion as *H. frisingense*. Similarly, the genome of strain cluster_DBSCAN_round4_6, previously identified as *H. frisingense*, also presented an ANI value below the 95% identity cutoff. Thus, our subsequent analyses included only the six genomes that met the cutoff for consideration as *H. frisingense* (ANI > 95%) (Figure 1). Within this group, four environmental isolates were found, GSF30, IAC152, BH-1, and SG826, and two clinical isolates, AU14559 and VT-16–41. Interestingly, in the ANI analysis, the two clinical strains clustered with the environmental strain GSF30.

### 2.3. Identification of Genomic Islands in H. frisingense AU14559

**Figure 2:**
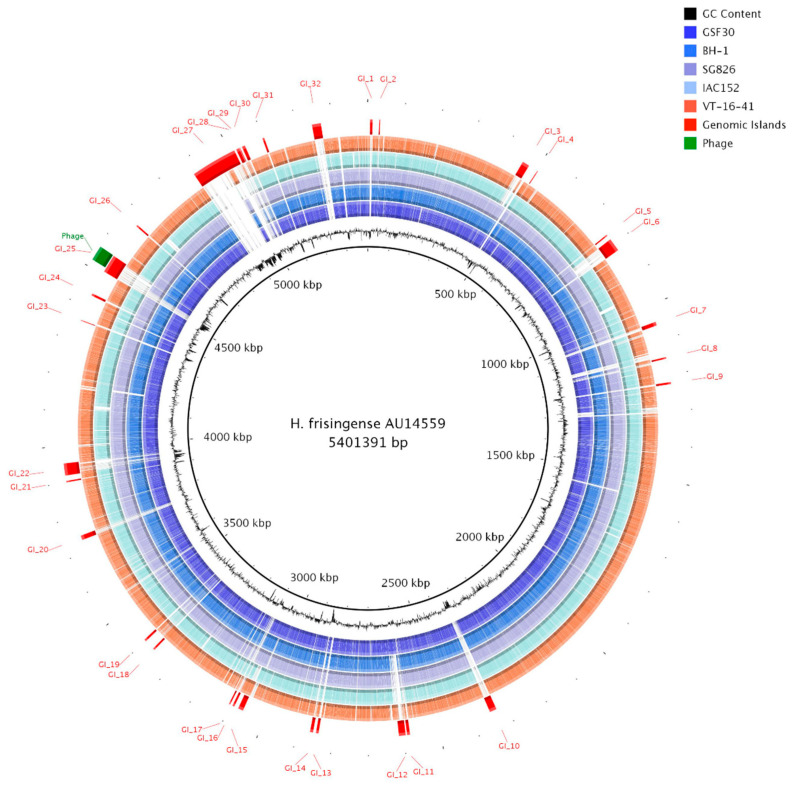
Circular representation of the genome of *H. frisingense* AU14559. The inner ring represents the GC content of the genome of *H. frisingense* AU14559. The next 5 rings represent the presence or absence in the other strains identified as *H. frisingense* of genes that encode proteins orthologous to those found in the AU14559 genome through alignments performed with BLASTP. The GI_1–GI_32 tags represent the genomic islands found by the AlienHunter and GIPSy algorithms, and the green region represents the possible phage sequence found by PHASTER.

The genomic comparison performed in this study suggests that there are no major differences in the genomic contents of the clinical and environmental strains. To further investigate the particularities of the genome of strain AU14559, we performed a genomic island analysis. Genomic islands are genomic regions that show some evidence of horizontal gene transfer, such as the absence of these sections in phylogenetically close individuals or pattern differences such as abnormal GC content and codon usage compared to the rest of the genome. Furthermore, the presence of genes related to horizontal gene transfer, such as genes that encode secretion systems and phage genes, can also be used as proxies for the identification of genomic islands [20,21]. The identification of possible regions arising from horizontal gene transfer was performed using the GIPSy and AlienHunter algorithms. Through the search performed by GIPSy, in which the genome of the strain *H. frisingense* IAC152 was used as a reference, we found a total of 14 genomic islands. GIPSy classified six of these islands (islands 3, 6, 8, 14, 25, and 32) as possible pathogenicity islands, five (islands 3, 6, 12, 19, and 20) as symbiosis islands, and three (islands 8, 20, and 27) as metabolic islands (Figure 2). Although this program also classifies islands associated with resistance, no genomic islands were classified in this category. The AlienHunter algorithm identified a total of 52 genomic islands in strain AU14559; however, most of these islands overlapped with each other or with the islands found by GIPSy. Therefore, the results of the two searches were merged, and overlapping island markings and regions that were also found in the other genomes of the species were excluded, resulting in a total of 32 possible genomic islands, GI_1 to GI_32 (Figure 2). Some of the predicted genomic islands were shared by all the studied representatives of the species, others were identified only in clinical isolates, and some were identified exclusively in AU14559.

A unique genomic island of AU14559 was GI_25, which is classified as a pathogenicity island. This island was about 46 kb and contained genes related to the type 6 secretion system (T6SS). Island GI_30 was approximately 15 kb in size and was also classified by GIPSy as a pathogenicity island. On this island, genes related to iron acquisition were found that did not have homologs in other *H. frisingense* strains, such as the genes encoding TonB (AU_47470 and AU_47550), ExbD (AU_47480), ExbB (AU_47480), hemolysin (AU_47500), heme binding protein (AU_47520), FecR (AU_47530), and FecI (AU_47540). When looking for common proteins among clinical isolates of the genus *Herbaspirillum* isolated from sputum from patients with cystic fibrosis, we found two islands that were shared with other isolates. Within island GI_26, there was a sequence that encoded 21 proteins (AU_44350-AU_44550); this sequence was also present in the clinical strain *H. seropedicae* AU14040 [7] but absent from the other strains of *H. frisingense* and *H. seropedicae*. These proteins are mainly involved in nucleotide metabolism and in the degradation of phenolic compounds through the protocatechuate pathway. We also identified island GI_7 representing nine proteins (AU_09530-AU_09610) that were shared with the isolate *H. seropedicae* AU13965; these proteins are mainly involved in energy metabolism.

Other than these two islands, the protein encoded by the AU_35290 gene that is present in island GI_22 was the only one shared (with an identity of ~70%) among the three clinical *Herbaspirillum* isolates; however, no genes encoding homologs of this protein were present in the genomes of the other strains of *H. seropedicae* and *H. frisingense*. This protein contains the peptidase C39 family domain, which is normally associated with the degradation of bacteriocins. To identify the bacteriocin related to this protein, a search for potential regions related to the production of bacteriocins in the genome was performed using the BAGEL4 algorithm [22]. However, no potential region of interest was found. It is possible that these genes were recently acquired from the bacterial community from which these strains were isolated. We also identified an insertion of approximately 40.2 kb originating from the phage genome and located within genomic island GI_25. This region was also present, with a coverage of between 53 and 58% in the genomes of five environmental strains of the species *H. seropedicae*, *H. rubrisubalbicans*, and *H. robiniae*, which show identities ranging from 81.2% to 87.4%.

### 2.4. Identification of Core and Accessory Genomes Using Clustering Analysis

**Figure 3:**
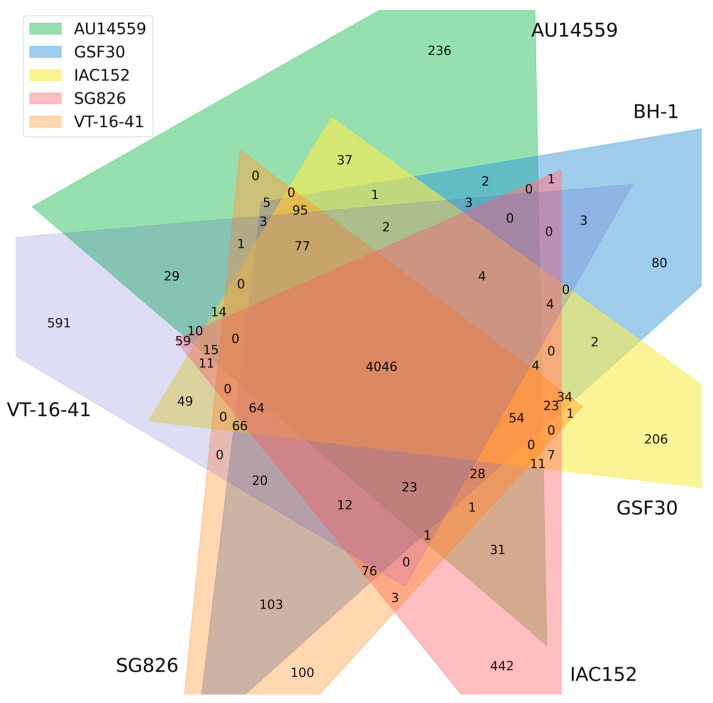
Analysis of the pangenome and core genome of *H. frisingense*. Venn diagram representing the homologous genes (identity > 50%) among genomes according to pan and core genome analysis performed by Panaroo. Each color in the graph represents a set of genes present in each genome. The numbers located in the non-overlapping regions between the polygons represent the total number of unique gene clusters in each strain. The numbers located in the overlapping regions between the polygons represent the total number of gene clusters identified by Panaroo that are shared among these strains. The central region of the graph represents the total cluster shared among all strains of *H. frisingense*; this cluster represents the core genome of this species.

The clinical strains of *H. frisingense* were isolated from sputum (AU14559) and urine (VT-16–41). These habitats were very different from the habitats from which environmental strains have been isolated, such as soil, rhizosphere, and plant tissue. This drastic difference in environments suggests, in principle, that these strains have undergone genomic adaptations that enable them to survive in different habitats. To identify the genes present in all individuals (core genome) and those unique to each strain (accessory genome), we compared the genomes of the six *H. frisingense* isolates with each other using the Panaroo program, a graph-based tool that uses CD-Hit, together with a neighborhood analysis, to divide the genes present in genomes into different groups according to their identities [23]. Of the 6690 different clusters created by Panaroo, 4046 were shared among all strains of *H. frisingense* (Figure 3). Strain AU14559 had a total of 236 unique clusters, many fewer than the 591 unique clusters in VT-16–41, the other clinical strain, and the 442 unique clusters of the environmental strain IAC152. The number of clusters shared between the two clinical strains, AU14559 and VT-16–41, was 29.

Among the genes identified as part of the core genome were some that could act in the process of colonization of both plants and humans, such as the cellulose biosynthesis operon (*wss*) and the cyclic b-glucan biosynthesis gene. The *wss* operon (AU_11910-AU_11990), composed of nine genes, was found in all genomes, and encodes proteins related to cellulose biosynthesis [24]. Bacterial cellulose is an important component that is needed for biofilm formation and, consequently, for host colonization by both plant and human pathogens [24,25]. Mutations in these genes can lead to deficiencies in biofilm formation [24]. Biofilm formation is important for the survival of pathogenic bacteria in the human body as it protects the bacteria against immunological defenses during host infection and promotes antibiotic resistance [26]. The National Institutes of Health (NIH) estimates that the percentage of human infections related to biofilm production caused by microorganisms is between 65% and 80% [27].

The AU_35540 gene encodes cyclic beta-1,2-glucan synthase; although the nucleotide sequence of this gene displayed low identity (<50%) with its homologs, the amino acid identity of the encoded protein with its counterparts in the other genomes of *H. frisingense* remained above 85%. This suggests that there is selective pressure for its functionality. This protein is responsible for synthesizing cyclic glucans, which have a cyclic homopolysaccharide structure formed by D-glucose molecules [28]. These molecules appear to act extracellularly to modulate the responses of animal and plant hosts [29].

Although many genes present in strain AU14559 had homologs in GSF30, some of the genes acquired by this strain are not present in environmental strains. Using kofamKOALA [30], we identified the metabolic pathways composed exclusively of genes found in the clinical isolate AU14559. We identified eight genes involved in the type VI secretion system (T6SS); these are annotated as 3 *vgrG*, *hcP*, *vasD*, *vasK*, *vasF*, and *clpV* (AU_45300, AU_45420, AU_45540, AU_45470, AU_45510, AU_45350, AU_45530, and AU_45430, respectively). This region was the same as described above for GI_25. In the analysis performed against the VFDB database, the *clpV* gene was also identified as a virulence factor unique to AU14559. However, when aligning the protein sequences of these genes, we observed that only the three proteins annotated as VgrG did not have homologs in the other strains of *H. frisingense*; the remaining five proteins were also found in the other strains, but with a protein identity of approximately 80%. The three AU14559 *vgrG* genes were closer in the genome in a 46,100 bp region but represented three distinct proteins (Figure S1). Although *vgrG* genes were not present in other *H. frisingense* strains, they shared high identity (~85%) with proteins found in the isolates of *H. seropedicae*. In the T6SS structure, the VgrG protein is a phage tail spike-like protein located at the outer end of the tube formed by the Hcp proteins, which facilitates penetration into neighboring cells [31]. All the analyzed strains possessed the type I secretion system Sec-SRP and Tat (twin-arginine translocation) and did not possess the type IV secretion system.

### 2.5. Plant Growth-Promoting Genes

**Figure 4:**
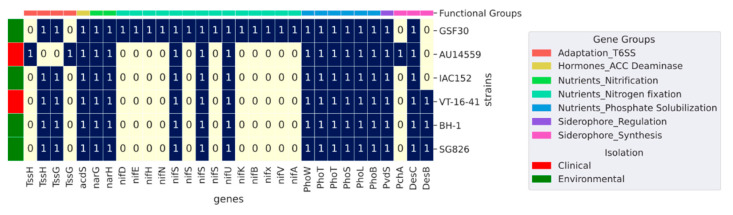
Plant growth-promoting factors identified through comparison via BLASTP between representatives of the clusters generated by Panaroo and the plant growth-promoting proteins listed by Kuramae et al. (2020) [18]. Only alignments that presented at least 80% coverage and 50% identity with the respective reference proteins were considered. Lines are colored according to isolation source, environment, and clinic, and the columns are colored according to the functional group to which each gene belongs.

*H. frisingense*, similar to *H. seropedicae* and *H. rubrisubalbicans*, has mechanisms to promote plant growth, including biological nitrogen fixation [4]. To identify the genes related to these mechanisms, we searched the genomes of *H. frisingense* strains for genes related to plant growth-promoting as listed by Kuramae et al. (2020) [18]. This database presents a set of bacterial genes related to interaction with plants, such as nitrogen fixation, nodulation, and phytohormones production. In addition, it has genes that can help bacterial colonization not only in animals, but also in plants, such as siderophore biosynthesis and secretion systems. Thus, this database was used to ascertain the content of these genes in the analyzed genomes. The nitrogenase enzyme complex, encoded by *nifHDK* genes, is responsible for the conversion of atmospheric nitrogen (N_2_) into ammonium (NH^4+^), making it assimilable by plants [4]. From the search of the database, we observed that only strain GSF30 possessed the genes that encode these enzymes; they were absent from all other strains, both clinical and environmental (Figure 4). On the other hand, all strains possessed *narGH* genes, allowing them to use nitrate as the final electron acceptor during oxidative phosphorylation [32]. Similarly, all strains possessed a copy of the *acdS* gene (1-aminocyclopropane-1-carboxylate deaminase), which encodes the protein responsible for the conversion of 1-aminocyclopropane-1-carboxylate to ethylene, an important phytohormone that is related to the increase in biomass [33]. The *phoBLS* and *pstABC* operons, which are associated with the solubilization and transport of phosphate, respectively [34,35], were also present in all strains.

### 2.6. Host–Pathogen Interaction

**Figure 5:**
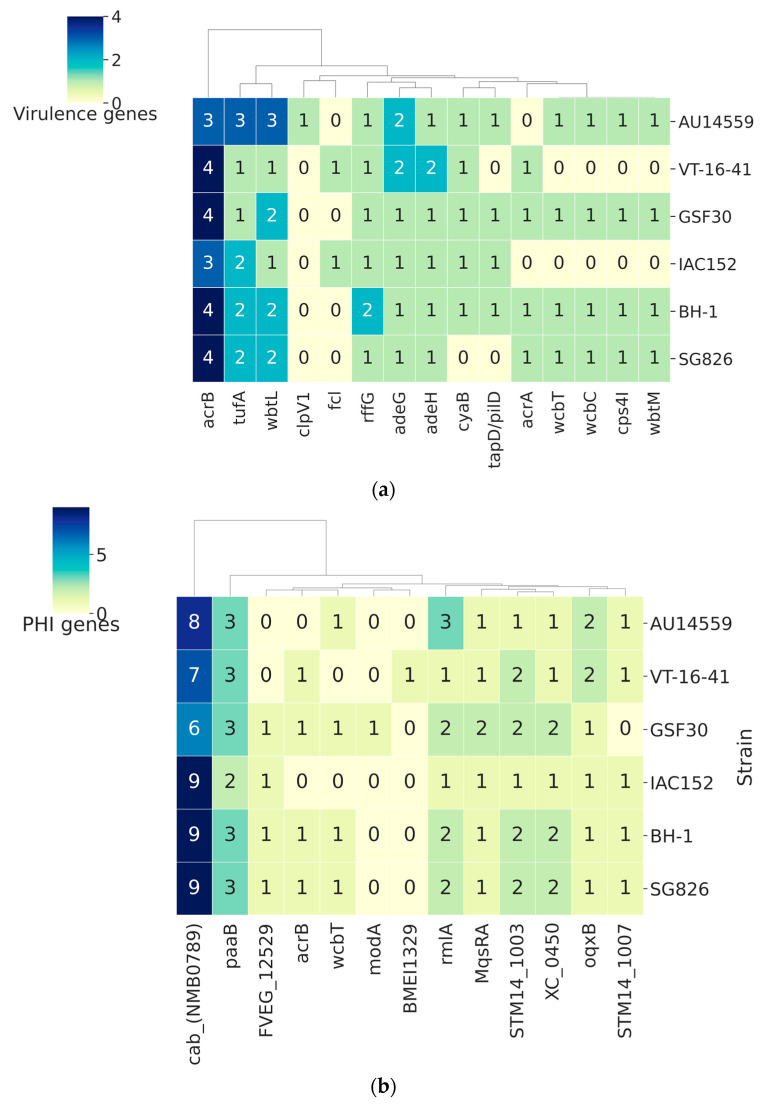
Interaction gene clustermaps: (**a**) virulence and (**b**) host–pathogen interaction factors found in *H. frisingense* strains. The identification was performed using BLASTP to compare representatives of the clusters generated by Panaroo with the proteins in the PHI-base and VFDB databases. Only sequences that displayed a minimum of 80% coverage and 50% identity when aligned with the proteins represented in the databases were considered.

An essential requirement for the colonization of a host, be it beneficial or pathogenic, is the interaction between the two organisms. To identify proteins that might be related to host–pathogen interactions, we subjected the predicted proteomes of the five strains to comparison with a database of virulence factors, VFDB (Figure 5a), and a database of interaction factors, PHI-base (Figure 5b). The clinical strain *H. frisingense* AU14559 presented a total of 73 virulence genes and was the strain with the highest number of genes in this category. However, among the virulence factors found in this analysis, only the AU_45430 gene, which aligned with the *clpV1* gene from the VFDB database, was unique to AU14559. The ClpV1 protein participates in the disassembly of the injection system that forms part of the type VI secretion system (T6SS) [36]. In the genome of *H. frisingense* AU14559, the T6SS region, in which the *clpV1* gene is inserted, was 46,100 bp in length and encoded 30 proteins. A comparison of this entire genomic region with the results obtained using the GenBank database showed that its highest level of identity was with the clinical strain *H. seropedicae* AU14040. *H. frisingense* IAC152 appeared as only the tenth best hit (Figure S1). The region that includes *clpV1* is the region that displays the lowest degree of DNA–DNA homology between AU14559 and other genomes of the genus *Herbaspirillum*. The ClpV1 protein from AU14559, in turn, showed 92.7% identity with ClpV1 from AU14040 and only 80% identity with ClpV1 from *H. frisingense* VT-16–41, the best hit within the species. Taken together, these results suggest that a recombination within the T6SS region resulted in the exchange of *clpV1* for a different version of the gene. Interestingly, strain VT-16–41, which is also pathogenic, presented only 67 genes that encode possible virulence factors and was the strain with the second-lowest number of genes in this category; moreover, unlike AU14559, this strain did not present any unique virulence factor. The other tested strains of *H. frisingense* (GSF30, BH-1, SG826, and IAC152) presented 70, 72, 69, and 65 possible genes related to virulence in their genomes, respectively, but these differences primarily reflect the differences in copy number (Figure 5a).

A feature of the high relevance for interaction with the host is the type of exopolysaccharide (EPS) produced. In *H. frisingense* GSF30, the probable O-antigen biosynthesis locus (OGC) has been identified and includes genes related to colitis synthesis [37]. In *H. frisingense* AU14559, the OGC was found in the region between 455,968 and 481,635 bp. Analysis of the nucleotide composition of the OGCs of GSF30 and AU14559 showed a DNA–DNA identity of 98.9% and identical gene compositions. However, studies of the O-antigen structure of different *Herbaspirillum* species and strains revealed that clinical strains AU14040 and AU13965 of *H. seropedicae* and strain AU14559 of *H. frisingense* do not contain rhamnose, unlike environmental strains [38]. To understand this phenotype, we located the *rfaBrmlABC* operon, which is responsible for the synthesis of rhamnose, in the genomes of clinical and environmental strains of *H. frisingense*. In that analysis, we found that the second gene in the operon, identified as *rmlA*, was mutated, creating a premature stop codon (Figure S2). Although the *rmlBC* genes were intact, it is possible that this mutation inactivates the entire rhamnose biosynthesis operon. In BH-1, we also identified a mutation, but in this case, the mutation was in the first gene of the operon. The other strains, including the VT-16–41 clinical strain, possessed the complete operon.

Through alignments with the PHI-base database, we identified a total of 199 genes in *H. frisingense* AU14559 that may be involved in host–pathogen interactions; however, none of these genes were found exclusively in this strain. The genomes of VT-16–41, IAC152, BH-1, GSF30, and SG826 presented 195, 194, 197, 195, and 197 occurrences, respectively, against proteins in this database (Figure 5b).

### 2.7. Antibiotic Resistance Genes in H. frisingense

**Figure 6:**
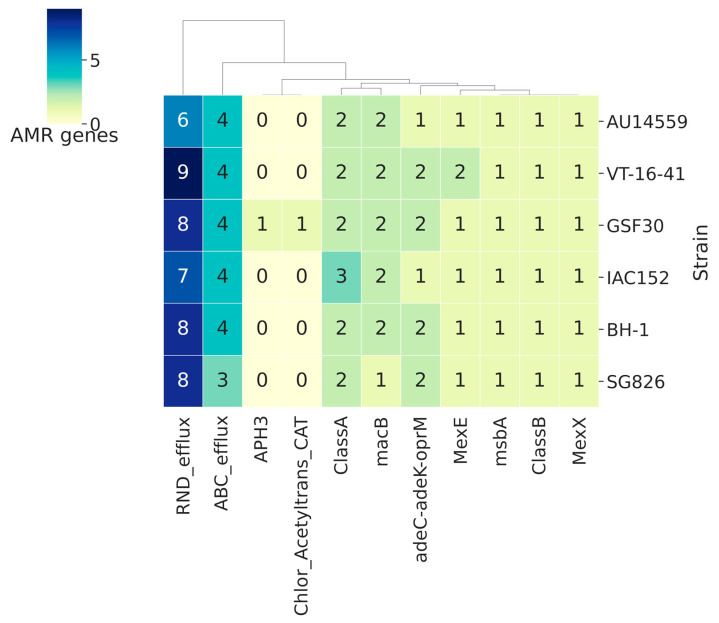
Clustermaps of the antibiotic resistance genes found in *H. frisingense* genomes. A representative of each cluster generated by Panaroo was compared with the ResFams database using the HMM ResFams core model. The lines in the clustermap represent the strains, and the numbers in the squares indicate the number of genes in that column found in the strain.

Antibiotic resistance is a feature that has become increasingly common in clinical isolates. Many resistance genes have already been mobilized in plasmids and are thus able to circulate among species in a hospital environment [39,40]. To identify genes potentially involved in antibiotic resistance mechanisms, we compared representatives of each of the clusters obtained by Panaroo from each of the five strains with the HMM profile of cured proteins from the Resfams database (core v1.2). Strain AU14559 possessed a total of 19 genes that are potentially involved in resistance (Figure 6). Strains VT-16–41, IAC152, BH-1, GSF30, and SG826 possessed 24, 21, 22, 24, and 20 proteins, respectively, that are potentially involved in antibiotic resistance. The main mechanism of action of resistance proteins encoded by the genome of strain AU14559 was drug efflux; its genome encoded nine RND efflux proteins (RND efflux, adeC-*adeK*-oprM, *mexE*, and *mexX*) and seven ABC efflux proteins (ABC efflux, *macB* and *msbA*). All the analyzed strains had 2 class A beta-lactamase genes belonging to the same cluster and a 1 class B beta-lactamase gene. In addition to these beta-lactamase genes, strain IAC152 had one additional class A beta-lactamase gene that was in a different cluster according to Panaroo’s analysis; this demonstrates that this gene is not an extra copy of another gene but that it encodes an enzyme that differs from the enzymes present in the other genomes of the species. Likewise, strain VT-16–41 possessed two genes that encode RND efflux proteins (RND_efflux and *mexE*), an MsbA protein that is involved in ABC efflux; however, the genes that encode these proteins were divided into two clusters by Panaroo. One of the clusters was present in VT-16–41 and IAC152, and the other was present in the other strains.

The results obtained in this analysis, as well as the results of the analysis of virulence genes, showed a very similar profile among the strains; in most cases, the strains varied only in the number of proteins in each family present. The exceptions were two unique genes present in the GSF30 environmental strain, those encoding aminoglycoside phosphotransferase (APH3) and those encoding chloramphenicol acetyltransferase. It is notable that there are many resistance genes associated with this species, even considering the environmental strains.

The presence of resistance genes in both clinical and environmental strains of *H. frisingense* raised the hypothesis that this is a common feature of the genus *Herbaspirillum*. To answer this question, we submitted all genomes of the genus *Herbaspirillum* (*n* = 76) to the same analysis performed for *H. frisingense*. The result of comparing these genomes with Resfams revealed that the antibiotic resistance genes found in *H. frisingense* were dispersed across the genus (Figure S3). In some isolates, we identified classes C and D beta-lactamases, in contrast to *H. frisingense*, which only had classes A and B. Interestingly, the isolates that had beta-lactamases class C and D did not have class A and B. Additionally, we found several genes identified as *vanS*. The *vanS/R* genes conditioned a two-component system that acted on vancomycin resistance, where VanS encodes a transcriptional activator.

## 3. Discussion

The emergence of new, opportunistic pathogens is a serious public health problem. The isolation of bacteria, which were initially described as environmental, in clinical samples such as blood, urine, and sputum has become increasingly common and distributed in different countries (Figure S4). Among these emerging bacteria, the genus *Herbaspirillum* makes a considerable contribution. Described in 1986 [2] as a bacterium that can endophytically associate with grasses such as rice and corn, in recent years several clinical strains of various species belonging to this genus have been isolated. *Herbaspirillum seropedicae*, *Herbaspirillum* *frisingense*, and *Herbaspirillum huttiense* are examples of species that have clinical isolates. In this work, we describe the sequencing and comparison of the genome of a new clinical strain of *Herbaspirillum frisingense* designated AU14559. The type strain of *H. frisingense*, GSF30, was described in 2001 as an environmental bacterium capable of biological nitrogen fixation [41]. Through our ANI analysis, we identified five genomes of the species *H. frisingense* that have been deposited in the GenBank database; four of these were environmental, and one was clinical. The search for genes related to biological nitrogen fixation was negative for all strains except the GSF30 type strain. Even the IAC152, BH-1, and SG826 strains, which were isolated from the environment, did not possess the genes necessary to perform biological nitrogen fixation. The high degree of identity among the proteins encoded by the *nif* clusters of *H. frisingense*, *H. seropedicae*, and *H. rubrisubalbicans* suggests that this cluster was acquired by an ancestral species via HGT [4]. The absence of the *nif* cluster indicates that this apparatus is still evolving and is not fixed in the species. Loss of the *nif* cluster has also been described in two other clinical isolates of *H. seropedicae* [42].

Exopolysaccharide (EPS) and lipopolysaccharide are important components of the bacterial cell membrane. In *H. seropedicae* it was shown that rhamnose has a high influence on the capacity of these bacteria to colonize maize roots. Rhamnose biosynthesis mutants showed a 100-fold reduction in adhesion and colonization [43]. According to Antunes et al., the clinical strains *H. frisingense* AU14559, *H. seropedicae* AU14040, and *Herbaspirillum* lineage 3 do not present rhamnose in their EPS, but all other species and strains analyzed do [38]. One possible hypothesis we suggest here is that this difference may be due to the action of the immune system. Park and Nahm showed that rhamnose is an important part of the immunodominant epitope in people immunized with the pneumococcal polysaccharide vaccine (PPSV23) [44], and Oyelaran and colleagues showed that human serum has a high titre of anti-alpha rhamnose antibodies [45]. Added to this is the fact that humans do not use or have rhamnose associated with their glycome [46]. It is possible that the bacteria that mutated and lost the ability to synthesize rhamnose, and therefore add it to their exo or lipopolysaccharide, were selected while the others were eliminated by an immune response.

The ability to acquire iron from the environment is extremely important for pathogenic bacteria. Iron acts as a co-factor in several metabolic reactions by coordinating enzymatic complexes. However, its bioavailability in the human body is low, and this makes it necessary for pathogens to maximize their ability to remove iron from the environment. In our study, we observed that the clinical strain AU14559 possessed additional genes for iron capture and transport, exemplified by TonB transporters. An important mechanism for extracellular iron capture is the production of siderophores, low-molecular-weight molecules with a high affinity for iron. Siderophores are synthesized by large enzyme complexes, usually those belonging to the non-ribosomal peptide synthetase group. Both clinical and environmental strains of *H. seropedicae* can produce siderophore serobactins. However, the gene encoding serobactin is not present in any strain of *H. frisingense*.

Core genome analysis revealed the presence of three *vgrG* genes unique to AU14559 among the genomes of *H. frisingense*. The VgrG protein is part of the T6SS and, together with PAAR, is responsible for the contact with neighboring cells [47]. A study conducted on *Serratia marcescens* showed that different VgrG homologues are associated with different effector proteins secreted by T6SS [48]. The region where these genes were identified was classified as a pathogenic island (GI_25). Despite not having a similarity with other *H. frisingense* genomes, GI_25 has a similarity with the genomes of clinical and environmental strains of *H. seropedicae*, with the clinical strain of *H. huttiense*, and with strains of *H. rubrisubalbicans*, considered a phytopathogen for some sugarcane crops [49]. The GI_25 is flanked upstream by a transponsase in addition to presenting regions with a sharp drop in GC content (Figure 2 and Figure S1). It is possible that these genomic features facilitate the recombination process.

The genome of *H. frisingense* AU14559 differs from that of the environmental isolate *H. frisingense* GSF30 in ways such as the loss of *nif* genes in addition to the acquisition of other genes. However, it also has several characteristics in common with the environmental strain that may be involved in its resilience in the host; these include the fact that the secretion systems of the two strains are identical, most of their virulence and interaction factors are similar, both possess genes such as the gene that encodes the protein responsible for the synthesis of cyclic beta-1,2-glucan, which is involved in the evasion of the human immune system and is a requirement for intracellular survival of other pathogens [50], and both possess the *wss* operon, which may be involved in biofilm production [51], an important strategy employed by pathogens for survival in the human body.

Antimicrobial resistance (AMR) in bacteria is a serious public health problem. According to the World Health Organization, AMR will be the leading cause of death worldwide in 2050 [52]. Antimicrobial resistance genes (ARGs) are increasingly common in the hospital environment [52], as is the identification of bacteria that have been previously described as environmental. In a previous study, we found that *Elizabethkingia* bacteria, initially isolated from soil, occupy the tenth position in the number of ARGs/genomes among bloodstream infection isolates [53]. Contrary to what might be expected, both clinical and environmental strains of *H. frisingense* share a high number of ARGs that are capable of conferring resistance to antibiotics of various classes. The resistance mechanism with the highest number of ARGs identified was drug efflux. Recently, our group carried out a study involving thousands of genomes of bacteria isolated from blood and we identified efflux pumps as one of the main resistance mechanisms [53]. However, the specificity of efflux systems still needs to be better elucidated. Some channels have known specificity to certain classes of drugs such as *macB*, which is an ABC transporter that confers resistance to macrolides [54], whereas RND transporters seem to have a lower specificity and are able to transport several classes of drugs [55]. An antibiogram analysis of the uropathogenic strain *H. frisingense* VT-16–41 (NCBI Biosample, accession number SAMN06130135) revealed that it is resistant to 11 different antibiotics. Considering that these genes are shared among different strains, it is possible that this profile extends to other strains. Antibiotic resistance was not deeply studied in the genus *Herbaspirillum* until the first clinical isolates emerged. However, when clinical strains began to be isolated and antibiogram tests began to be performed, it was possible to determine that the resistance profiles of these clinical isolates generally encompass more than three antibiotics of different classes; this classifies them as multidrug resistant (MDR) [56]. In addition, many of these clinical isolates of *Herbaspirillum* have intrinsic resistance against colistin, an antibiotic of last resort [5,14,57].

The microbiota present in the rhizosphere are extremely important in plant development; they influence nutrient acquisition, hormone production, and the defense against plant pathogens. This microbiota are also the main source of environmental bacteria for the human microbiome, modulating our own microbial diversity through food [58]. However, ingestion of these bacteria, together with raw vegetables, acts as a source of dissemination of resistance genes for humans, which are mainly derived from nonpathogenic bacteria present in the soil [58,59,60]. With the domestication of plants of economic interest, human action also influences the diversity of these microbiota and the functional composition of their genes, especially in organic farming, where sewage sludge and animal manure can be used as fertilizers [61,62] and where the number of resistance genes is significantly higher than that present in plants produced through conventional cultivation [63].

The results presented in this work are of particular importance considering that *H. frisingense GSF30*, as well as *H. seropedicae* and *H. rubrisubalbicans*, is widely used as a biofertilizer in grass crops [25]. We must evaluate the possibility that *H. frisingense* has the potential to colonize and infect a variety of hosts. This may be because the “native” environment of these bacteria, the rhizosphere, facilitates the emergence of opportunistic bacteria [64]. Cell adhesion and cytotoxicity studies with the environmental strain *H. seropedicae* SmR1 and the clinical strains *H. huttiense* spp. *huttiense* AU11883 and *H. frisingense* AU14559 showed that the clinical strains can adhere to lung Calu-3 cells. Furthermore, the treatment of Calu-3 cells with the strain AU14559 secretome led to drastic changes in cell morphology [65]. The rhizosphere is a nutrient-rich environment due to the exudates released by the plants, and this generates great competition between the bacteria present in this environment and thus acts as a selection factor for cells with more efficient nutrient and mineral uptake mechanisms; this is a common feature among human pathogens. This competitive environment often also makes it necessary for bacteria to develop defense mechanisms against other microorganisms, both through the production of defenses with the acquisition of antibiotic resistance genes and through the production of antibiotics and the infection of other eukaryotic organisms present in their environment, such as fungi and protozoa. These characteristics, together with the presence of genes that affect bacteria–plant interactions and may also be associated with bacteria–human interactions, may contribute to the recent cases in which *H. frisingense* has been identified in clinical samples.

## 4. Materials and Methods

### 4.1. Purification and Sequencing of Genomic DNA

*H. frisingense* AU14559 cells were grown overnight in NFbHP medium [66] at 37 °C until the culture reached an O.D. 600 nm of 0.8. Genomic DNA was purified using the phenol chloroform method [67], and the genome was sequenced in a hybrid strategy using the MiSeq paired-end 2 × 300 bp (Illumina Inc., Foster City, CA, USA) and Ion Proton single-end 1 × 200 bp (Thermo Fisher, Waltham, MA, USA) platforms.

### 4.2. Genome Assembly

The *H. frisingense* AU14559 genome was assembled individually and in a combined manner using the results obtained from the different sequencing platforms using Newbler v2.9 assemblers (454 Life Sciences, Branford, CT, USA), SPAdes v3.10.0 [68] and CLC assembler v10 (Qiagen). The assemblies were evaluated using the QUAST (Quality Assessment Tool for Genome Assemblies) algorithm [69] to choose the best automatic assembly. The contigs were then sorted using the genome of *H. frisingense* GSF30, the closest genome to strain AU14559, as a reference. As the genome of strain GSF30 was not closed, its contigs were ordered using the genome of *Herbaspirillum seropedicae* SmR1, the most closely related species with a closed genome, as a reference by visualizing the contigs in the Artemis Comparison Tool (ACT) [70]. Gap closure was performed using the algorithms FGAP [71] and GFnisher [72] and by manual inspection, looking for regions of similarity at the ends of the contigs and in assemblies performed by other assemblers using the BLASTN algorithm [73]. To validate the assembly, all reads were mapped onto the assembled genome with the purpose of identifying regions without coverage and inconsistencies in the genome. The closed genome was annotated using the Prokka algorithm that coordinates other tools to provide a complete genome annotation (ncRNA, tRNA, rRNA, and CDSs) [74]. The ncRNAs (non-coding RNA) were identified by Infernal using the RNA Families database (RFam) as reference and considering the RNA secondary structure.

### 4.3. Genome Comparisons

All publicly available genomes of the genus *Herbaspirillum* were retrieved and compared by calculating the average nucleotide identity (ANI) using the pyani pipeline [75]. Similarities and divergences were investigated using a genome–genome alignment performed using BLASTN [73] and visualized using ACT [70] and BRIG [76] software. The genes present in the clinical strain and absent from the environmental strains were further investigated with respect to their function, the domains present in their encoded proteins, and their relationship with interaction and resilience in humans. This process was performed using the BLASTP algorithm [77] and the Pfam database [78] and searches in PubMed. Possible horizontal gene transfer regions were determined by GIPSy software [79] using the genome of *H. frisingense* IAC152 as a reference and using Alien Hunter [80] without the use of a reference genome.

The genomes of the strains identified by the ANI calculation as belonging to *H. frisingense* were re-annotated by the Prokka algorithm [74] to standardize the CDS annotations. The resulting genes from each of these annotations, together with the genes from the *H. frisingense* strain AU14559, were then clustered using Panaroo [23] using a family threshold of 50% identity and length difference cutoff of 0.95.

To identify virulence and host–pathogen interaction factors in the analyzed genomes, the BLASTP algorithm [77] was used to perform an alignment of searches of the representatives of the clusters resulting from the analysis with Panaroo against the core dataset (only proteins verified experimentally) from the VFDB database (Virulence Factors DataBase) [81] and PHI-base (Pathogen Host Interactions database) [82]. For both searches in the databases, a cutoff of 50% identity, an e-value of 10-6, and a minimum coverage of 90% were used. Proteins from groups present exclusively in the clinical strain AU14559 were inserted into the KofamKOALA [30] tool to identify the metabolic pathways in which they are involved. Possible phage regions incorporated into the genome were identified using PHASTER [83]. To search for genes that confer resistance to antibiotics, the algorithm HMmer v3.3.2 [84] was used, searching for the proteins representing the clusters in an HMM profile of the cured bank of antibiotic resistance genes, ResFams (Core v1.2) [85]. Potential bacteriocin-coding regions were searched using BAGEL4 [22].

### 4.4. Prospecting for Plant Growth-Promoting Genes

From the list of genes compiled and classified by Kuramae et al. (2020) [18], CDSs in fasta format were programmatically extracted from the REST API of the UniProt database [86]. The created multifasta (amino acid) file was used to identify plant growth-promoting rhizobacteria genes (PGPRGs) (S-1). The CDSs were then aligned using the BLASTP program [77] against the Panaroo counterpart clusters. PGPRGs were considered as matches when they covered 80% of a cluster with an average identity of 50%.

## 5. Conclusions

Our results show that there are great similarities between the genomes of clinical and environmental strains. Regarding AMR, we found several antibiotic resistance genes distributed among all strains, including genes encoding class A and class B beta-lactamases. The antibiogram profile determined for the uropathogenic strain and the high identity among homologs in the different strains suggest that these genes are functional and that the profile is shared. Furthermore, we showed that all strains other than the type strain have lost the ability to fix nitrogen, but have retained other genes that are important for host interaction. Our data suggest that these bacteria present a pre-adaptation to different hosts and reinforce the notion that bacteria that interact with one host are more likely than free-living bacteria to colonize another host.

## Data Availability

The genome sequence of *H. frisingense* strain AU14559 has been deposited in GenBank under accession number CP083589. The genomes of the other strains can be retrieved using the corresponding accession numbers: *H. frisingense* GSF30 accession number AEEC02; *H. frisingense* IAC152 accession number NZ_CP049139; *H. frisingense* VT-16–41 accession number MUXB01; *Herbaspirillum* sp. SG826 accession number JAAOYU0; *Herbaspirillum* sp. BH-1 access number PKOI01.

## Supplementary Materials

The following are available online at https://www.mdpi.com/article/10.3390/antibiotics10111409/s1, Figure S1: Genomic region of *H. frisingense* AU14559 containing the type VI secretion system (T6SS), Figure S2: Schematic representation of the *rfbBrmlACD* operon identified in the genomes of *H. frisingense* strains, Figure S3: Antibiotic resistance genes found in *Herbaspirillum* genomes, Figure S4: Distribution of clinical isolates of the genus *Herbaspirillum* according to published papers.
