# Supplementary material for: High Genomic Identity between Clinical and Environmental Strains of Herbaspirillum frisingense Suggests Pre-Adaptation to Different Hosts and Intrinsic Resistance to Multiple Drugs
Source: Antibiotics (Basel). 2021 Nov 18;10(11):1409. doi: 10.3390/antibiotics10111409 (PMC8614823; doi:10.3390/antibiotics10111409)
Supplement: Supplementary file 1 [file antibiotics-10-01409-s001.zip › antibiotics-1415854-supplementary.pdf]

SUPPLEMENTARY FILE

High genomic identity between clinical and environmental strains of *Herbaspirillum frisingense* suggests pre-adaptation to different hosts and intrinsic resistance to multiple drugs

Willian Klassen Oliveira [1,2], Hugo Leonardo Ávila [1], Michelle Zibeti Tadra [3], Rodrigo Luis Cardoso [3], Cyntia Maria Teles Fadel-Pichet [4], Emanuel Maltempi de Souza [3], Fábio de Oliveira Pedrosa [3], Helisson Faoro [1,2,*]

1 Laboratory for Applied Science and Technology in Health, Instituto Carlos Chagas, FIOCRUZ Paraná. Algacyr Munhoz Mader Street, 3775, Curitiba 81350-010, Brazil; willianklasoli@gmail.com (W.K.O.); hugoavila@protonmail.com (H.L.Á.); helisson.faoro@fiocruz.br (H.F.)

2 Graduation Program in Biofinformatics, Universidade Federal do Paraná, Curitiba 81520-260, Brazil

3 Department of Biochemistry and Molecular Biology, Universidade Federal do Paraná, Curitiba 81531-980, Brazil; mzitadrasfeir@gmail.com (M.Z.T.); luiz.grabarski@gmail.com (R.L.C.); souzaem@ufpr.br (E.M.d.S.); fpedrosa@ufpr.br (F.d.O.P.).

4 Department of Clinical Analyses, Universidade Federal do Paraná, Curitiba 80060-240, Brazil; fpicheth@ufpr.br

* Correspondence: helisson.faoro@fiocruz.br or hfaoro@gmail.com; Tel.: +55-(41)-3316-3230

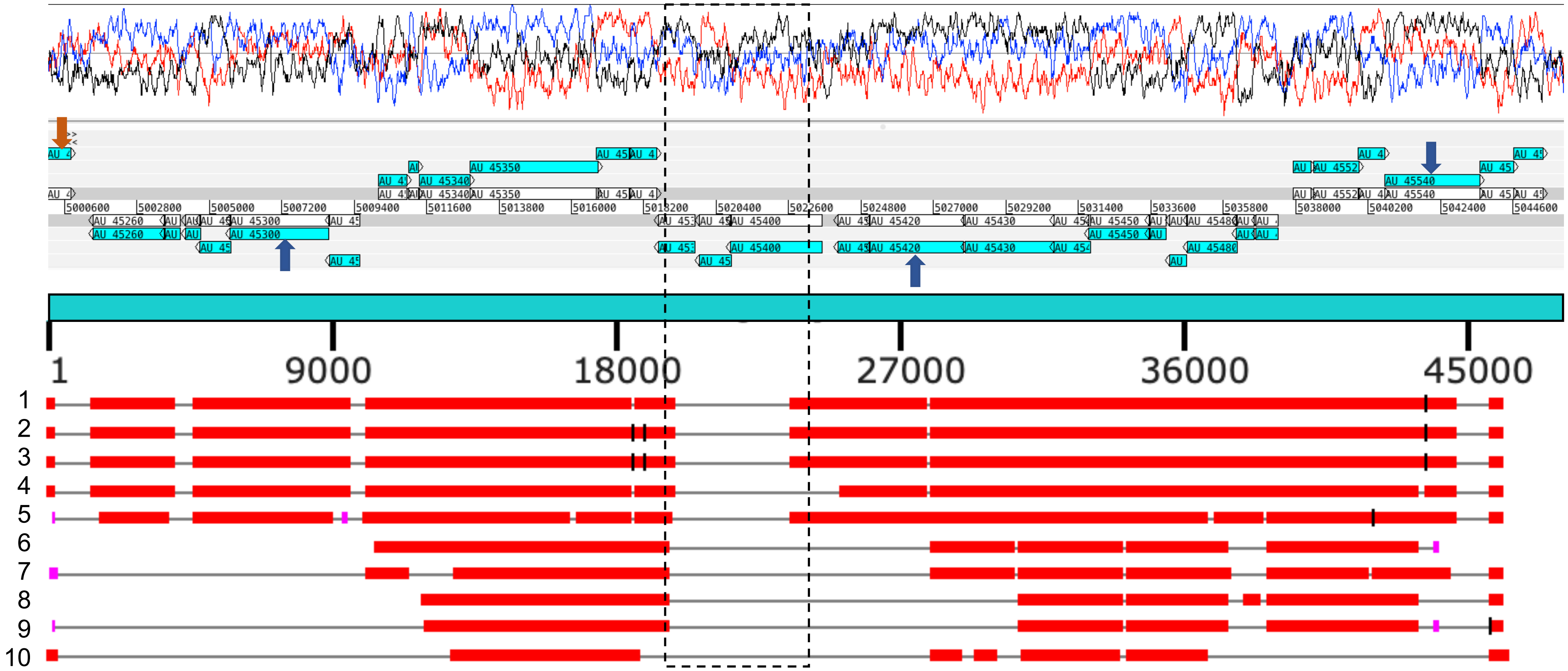

Figure S1: Genomic region *of H. frisingense AU14559* containing the type VI secretion system (T6SS). The upper part of the figure represents the GC% content in Framplot format. The cyan arrows represent the genes encoded in that region. The lower part represents the blastn alignment score in the following descending order of score: 1- *H. seropedicae* AU14040; 2- *H. seropedicae* Z67; 3- *H. seropedicae* SmR1; 4- *H. seropedicae* AU13965; 5-*H. robiniae* AA6; 6- *H. rubrisubalbicans* Os34; 7- *H. huttiense* NFYY53159; 8- *H. rubrisubalbicans* M1; 9- *H. rubrisubalbicans* DSM11543; 10- *H. frisingense* IAC152. The orange arrow indicates a transposase. The dark blue arrows indicate the 3 *vgrG* genes. The dotted box indicates the region of *clpV* that underwent recombination.

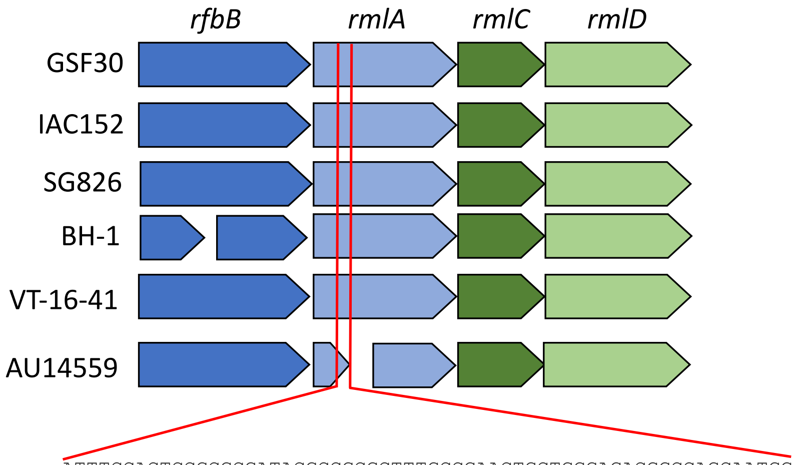


```
GSF30        ATTTCCACTCCGCGCGATACGCCGCGCTTTGCCGAACTGCTGGGAGACGGCCAGCAATGG  240
IAC152       ATTTCCACTCCGCGCGATACGCCGCGCTTTGCCGAACTGCTGGGAGACGGCCAGCAATGG  240
SG826        ATTTCCACTCCGCGCGATACGCCGCGCTTTGCCGAACTGCTGGGAGACGGCCAGCAATGG  240
BH-1         ATTTCCACTCCGCGCGATACGCCGCGCTTTGCCGAACTGCTGGGAGACGGCCAGCAATGG  240
VT-16-41     ATTTCCACTCCGCGCGATACGCCGCGCTTTGCCGAACTGCTGGGAGACGGCCGGCAATGG  240
AU14559      ATTTCCACGCCGCGCGATATGCCGCGCTTTGCCGAACTGCTGGGAGACGGCTAGCAATGG  240
             ******** ********** *******************************  *******
```

Figure S2: Schematic representation of the *rfbBrmlACD* operon identified in the genomes of *H. frisingense* strains. The highlighted region represents the multiple sequence alignment by ClustalW identifying the mutation responsible for the insertion of a premature stop codon in the *rmlA* gene of *H. frisingense* AU14559.

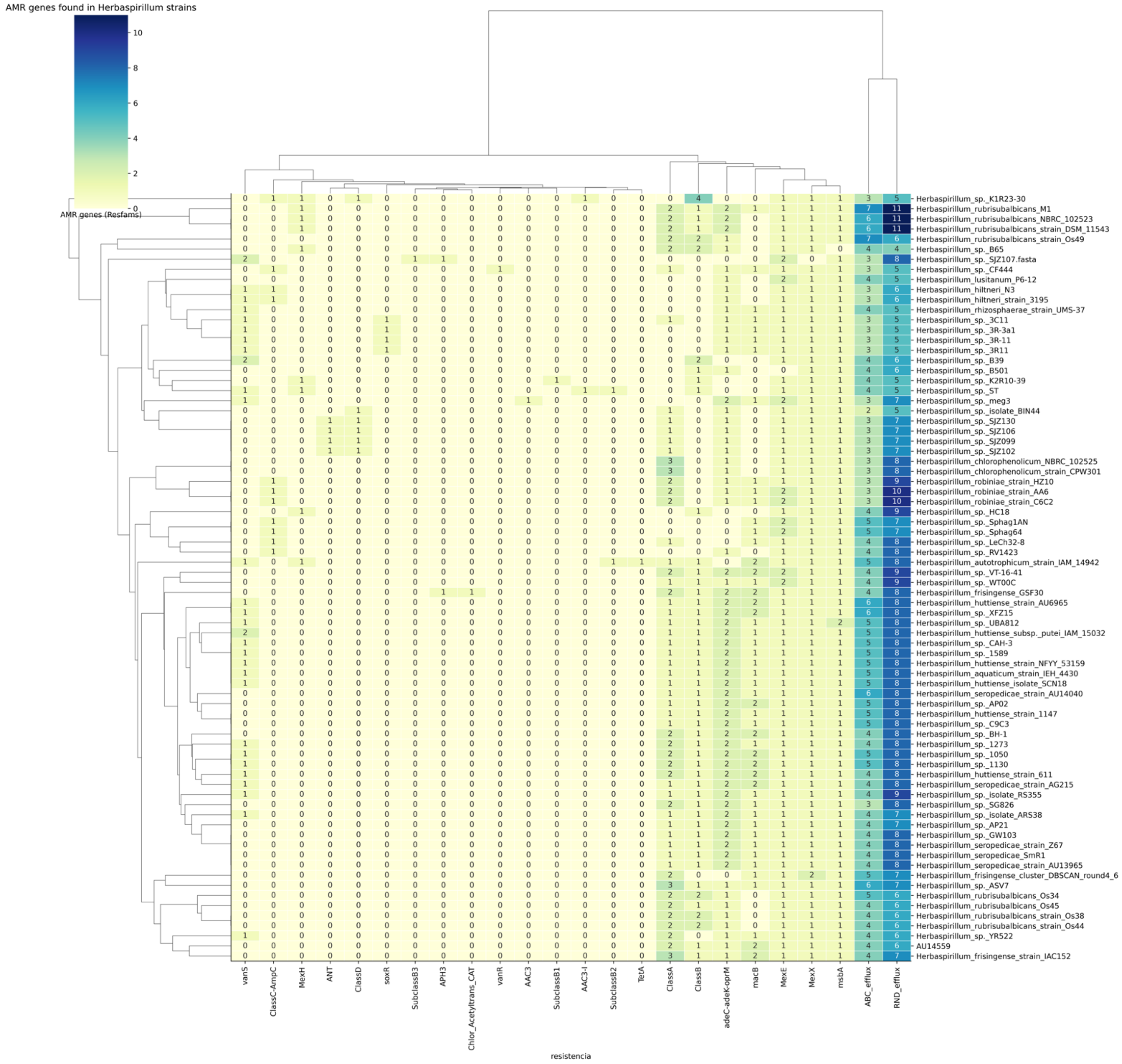

Figure S3: Antibiotic resistance genes found in *Herbaspirillum* genomes. The predicted proteome of each strain was compared with the Resfams database using the HMM Resfams core model. The lines in the heatmap represent the strains, and the numbers in the squares indicate the number of genes in that column found in the strain.

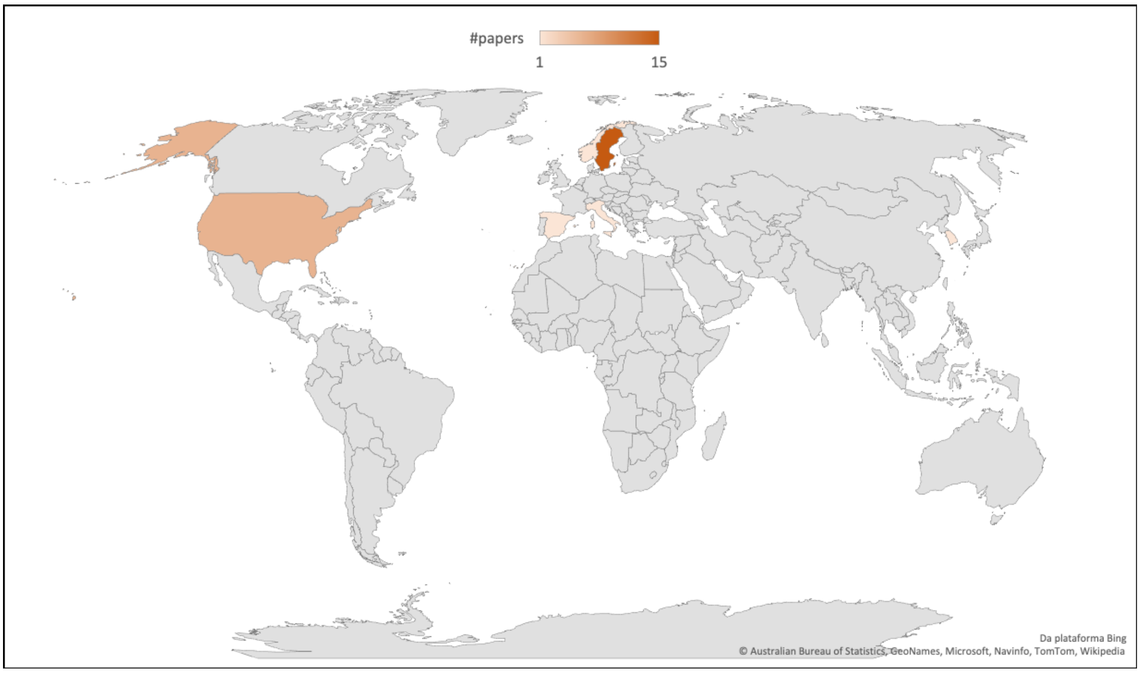

Figure S4: Distribution of clinical isolates of the genus *Herbaspirillum* according to published papers.
